# The Incidence of Human Cysticercosis in a Rural Community of Eastern Zambia

**DOI:** 10.1371/journal.pntd.0002142

**Published:** 2013-03-21

**Authors:** Kabemba E. Mwape, Isaac K. Phiri, Nicolas Praet, Niko Speybroeck, John B. Muma, Pierre Dorny, Sarah Gabriël

**Affiliations:** 1 Department of Clinical Studies, School of Veterinary Medicine, University of Zambia, Lusaka, Zambia; 2 Department of Veterinary Tropical Diseases, Faculty of Veterinary Sciences, University of Pretoria, Pretoria, South Africa; 3 Department of Biomedical Sciences, Institute of Tropical Medicine, Antwerp, Belgium; 4 Institute of Health and Society, Université Catholique de Louvain, Brussels, Belgium; 5 Department of Disease Control, School of Veterinary Medicine, University of Zambia, Lusaka, Zambia; 6 Laboratory of Veterinary Parasitology, Faculty of Veterinary Medicine, Ghent University, Merelbeke, Belgium; Universidad Nacional Autónoma de México, Mexico

## Abstract

A community-based longitudinal study was performed in the Eastern Province of Zambia, in which repeated serological samplings were done to determine the incidence of human cysticercosis. Three sampling rounds were carried out at six months intervals. A total of 867 participants presented for all three samplings. All samples were tested for the presence of cysticercus antigens using a monoclonal antibody-based enzyme-linked immunosorbent assay (sero-Ag-ELISA), while a randomly selected sub-sample of 161 samples from each sampling round was tested for specific antibodies using a commercial enzyme-linked immunoelectrotransfer blot (EITB) assay. Stool samples (n = 226) were also collected during the final round of sampling for taeniosis diagnosis by coprology and coproantigen ELISA. Cysticercosis seroprevalence varied from 12.2% to 14.5% (sero-Ag) and from 33.5% to 38.5% (sero-Ab) during the study period. A taeniosis prevalence of 11.9% was determined. Incidence rates of 6300 (sero-Ag, per 100000 persons-year) and 23600 (sero-Ab, per 100000 persons-year) were determined. Seroreversion rates of 44% for sero-Ag and 38.7% for sero-Ab were recorded over the whole period. In conclusion, this study has shown the dynamic nature of *T. solium* infections; many of the people at risk become (re)infected due to the high environmental contamination, with a high number turning seronegative within a year after infection. An important number of infections probably never fully establish, leading to transient antibody responses and short-term antigen presence.

## Author Summary

Human neurocysticercosis is an infection of the central nervous system caused by the larval stage of the pork tapeworm (*Taenia solium*). The infection occurs mainly in developing countries and is associated with poverty, poor sanitation and free-range pig management. It is estimated to be responsible for 30% of cases of acquired epilepsy in endemic areas. The limited number of human studies on this infection in Sub-Saharan Africa determined a high occurrence of cysticercosis. This study aimed to learn more about the transmission dynamics of this parasite in a rural endemic area in Eastern Zambia. A longitudinal study was carried out in which 867 participants were blood sampled three times, with a 6-month interval. Samples were analysed for the presence of cysticercal circulating antigens and specific antibodies. Results indicate that about 1 on 3 people get exposed to infection while only 1 on 10 people actually acquire infection. The study shows the dynamic nature of *T. solium* infections; many of the people at risk become (re)infected due to the high environmental contamination, with a high number turning seronegative within a year after infection. An important number of infections probably never fully establish, leading to short-term antibody and antigen presence.

## Introduction

Human (neuro) cysticercosis, an infection caused by the metacestode larval stage of the pork tapeworm *Taenia solium*, is a serious but neglected zoonotic disease and a major public health problem in many developing countries of Latin America, Asia and Africa [1], [2]. Humans are the definitive hosts harbouring the adult tapeworm (leading to taeniosis). Carriers of the tapeworm shed eggs into the environment that are infective not only to the pig intermediate host (leading to porcine cysticercosis) but also to humans who then act as an accidental intermediate host [3] leading to human cysticercosis. When the larval stages invade the nervous system they cause neurocysticercosis (NCC), which is the most important parasitic disease affecting the nervous system and accounts for about 30% of all acquired epilepsy cases in endemic areas [4]. In terms of Disability Adjusted Life Years (DALYs), the global burden of epilepsy is estimated at 7.8 million DALYs with 6.5 million of these occurring in *T. solium* endemic regions of the world [5].

The few community based human prevalence studies carried out in Africa have indicated sero-prevalences of human cysticercosis ranging from 7–22% [e.g. 6], [7], . In a recent study in Zambia, a sero-prevalence of 5.8% has been recorded in a rural community in the eastern part of Zambia [9].

Studies that report incidence of human cysticercosis are even more scarce and absent for Sub-Saharan Africa. Two longitudinal studies in villages in Peru indicated human cysticercosis incidence rates of 25% and 8% by specific antibody analysis [10]. In a simulation model based on data obtained in a rural community in Ecuador an annual incidence rate of 14% was described [11].

Obviously, more information is needed on the transmission dynamics of this parasite. The present study aimed at determining the incidence of human cysticercosis in an endemic area.

## Materials and Methods

### Ethical statement

The University of Zambia Biomedical Research Ethics Committee granted ethical clearance (IRB0001131) for the study. Further approval was sought from the Ministry of Health of Zambia, from the local district health authorities and the area chief. Meetings were held with the people in the villages through their leaders (headmen) to explain the purpose of the study, request their permission to conduct the study and also to invite them to participate. Participation was requested of individuals of all ages after written informed consent. For individuals below the age of 16, permission was sought from their parents or guardians by way of written informed consent. All participants found positive for taeniosis and other helminths were provided with treatment, namely niclosamide and mebendazole respectively. Those positive for cysticercosis were referred to the District hospital for follow-up and the recommended standard of care provided to them if required.

### Study area and population

**Figure 1:**
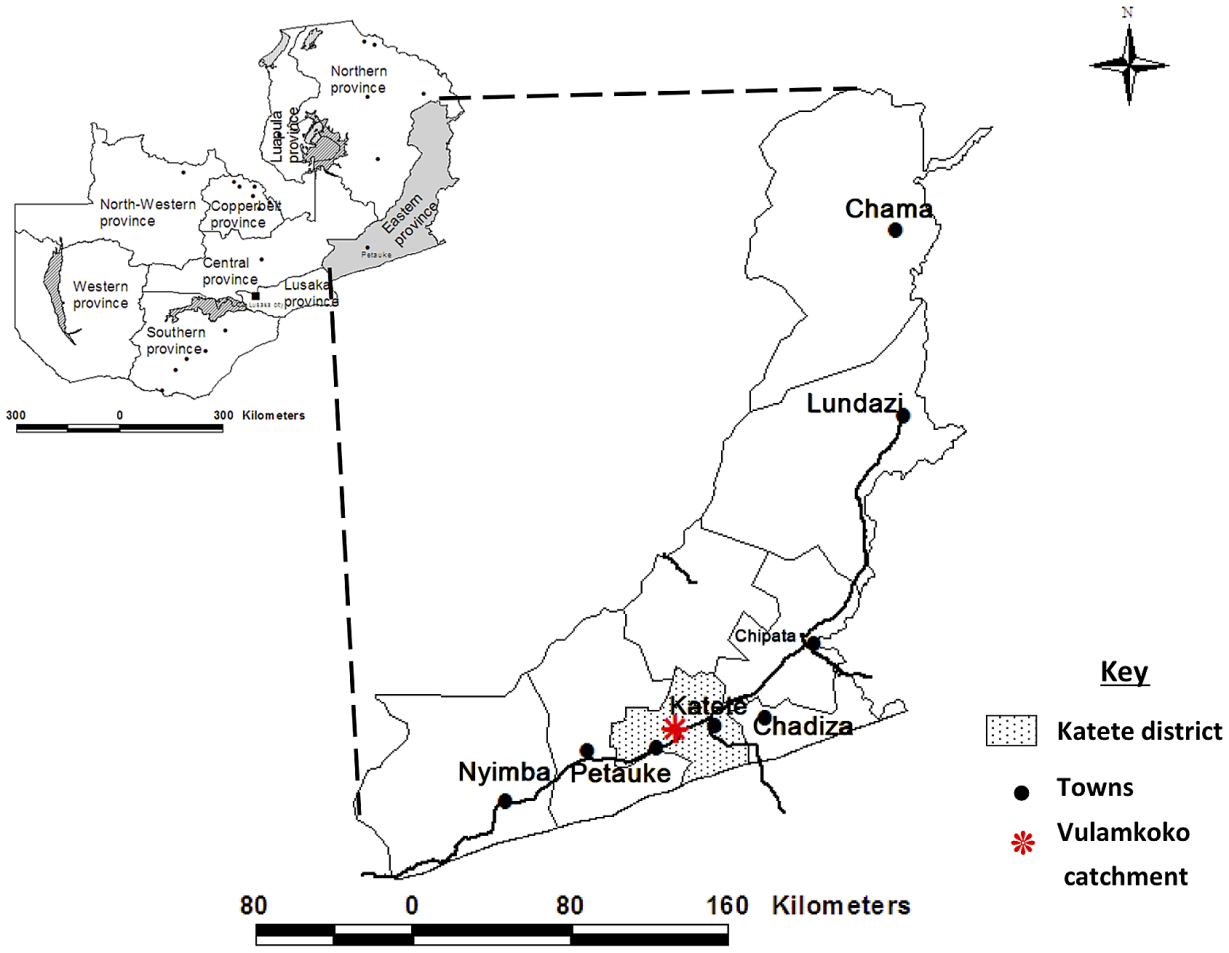
Map of Zambia showing the study area (Vulamkoko catchment) in Katete district of the Eastern province.

The study was carried out in the Vulamkoko community in Katete district of the Eastern province of Zambia (figure 1). The Vulamkoko Rural Health Center (RHC) provides health care in this community with a catchment population of 23,613 (clinic headcount records). The climate is tropical with two main seasons, the rainy season (November to April) and the dry season (May to October/November). The mean rainfall varies from 500 to 1200 mm/year with temperatures above 20°C most of the year. The most common ethnic group in Katete is the Chewa people. They practice subsistence agriculture raising animals and growing crops. People's homes in this area are of adobe and have no sanitary facilities. Pigs have access to the nearby bushes that are used as latrines by the villagers.

The community was selected for the study on the basis of known endemicity for porcine cysticercosis [12], presence of free roaming pigs, backyard slaughter of pigs without meat inspection, continued observation of cysticerci in the meat, absence of any cysticercosis related control programs and the community's willingness to participate. All willing villages within a radius of 7 km from the RHC were selected. The willingness of the RHC to collaborate, and the availability of staff and adequate working space was also taken into account.

### Study design

**Figure 2:**
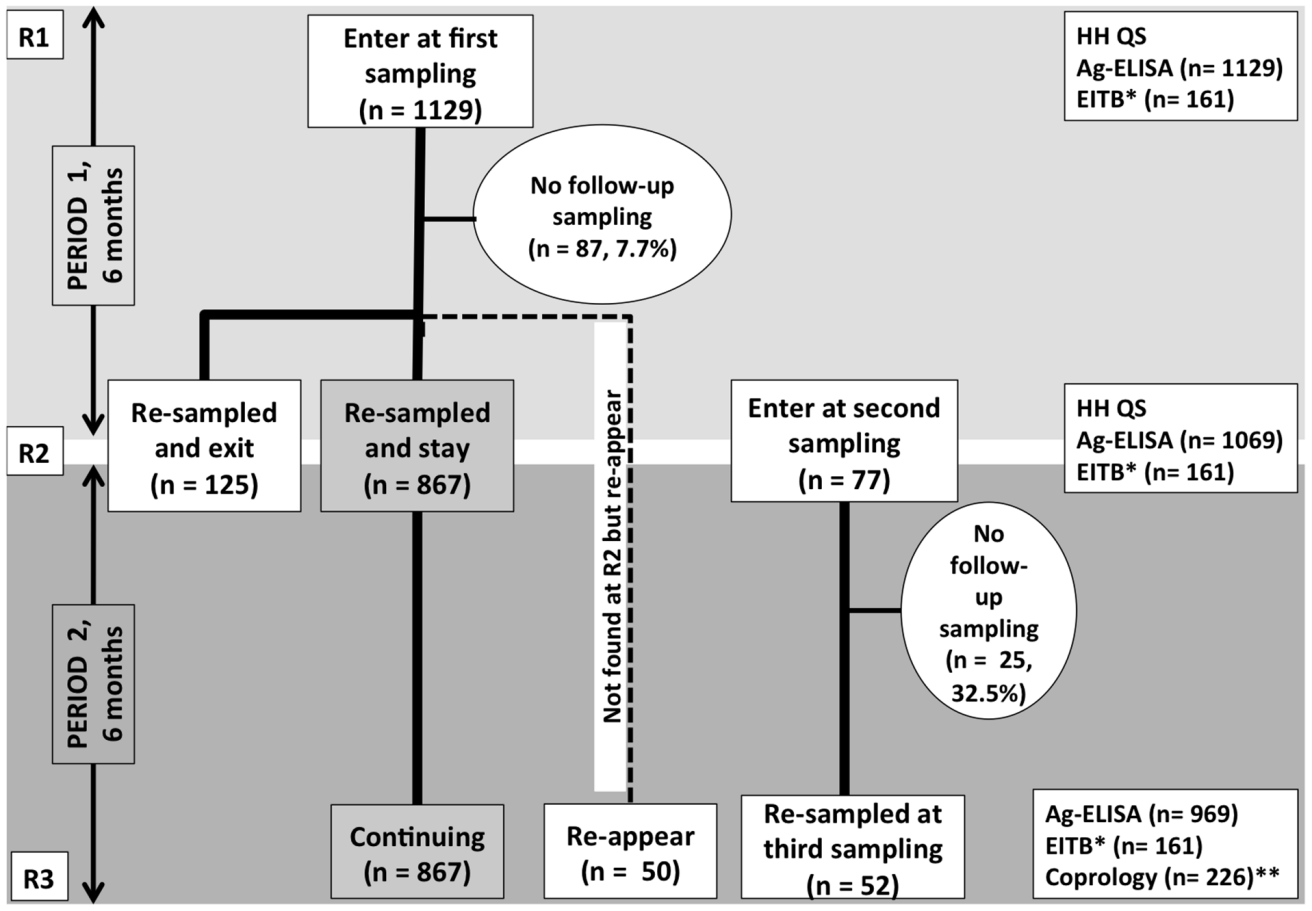
Cohort composition and changes during the three sampling rounds. (Format adapted from Garcia *et al*., 2003 [18]). R1, R2 and R3 stand for first, second and third round of sampling. Ag-ELISA: detection of circulating cysticercus antigen in serum; EITB: detection of specific antibodies in serum; HH QS = household questionnaire; Coprology: coproscopy and Copro-Ag-ELISA. *Only carried out on a randomly selected sample of 161. **Carried out on stool samples collected at R3.

A community-based longitudinal study was carried out between October 2009 and October 2010, with three main sampling rounds (R1, R2, R3) with six months intervals (figure 2). Participants who were not sampled in the first round of sampling and willing to participate were entered in the study only during the second round of sampling.

Meetings were held in the selected villages and individuals of all ages of all households invited to participate in the study. The sampling unit was an individual in a household. Each willing participant, after written informed consent, was registered and had a blood sample taken by qualified health personnel every six months during a 12-month period (a total of 3 samples). During the last sampling round, a stool sample was also requested from the participants.

A questionnaire was administered to each participating household to obtain information on general household characteristics, pig management and sanitation (Mwape *et al.*, submitted).

### Sample collection and analyses

About 5 ml of blood were collected, serum extracted, aliquoted and stored at -20°C until use. Submitted stool samples were divided into two aliquots, one placed in 10% formalin and the other in 70% ethanol and stored until use. All the samples were transported to Lusaka for analysis [9].

The serum samples were tested for circulating cysticercal antigens using the monoclonal antibody-based B158/B60 antigen enzyme linked immunosorbent assay (sero-Ag-ELISA) as described by Dorny *et al.* (2004) [13]. To determine the test result, the optical density of each serum sample was compared with a series of 8 reference negative human serum samples at a probability level of *P*<0.001 [13].

Due to budgetary restrictions, not all samples could be analysed for presence of specific antibodies. Therefore, from the individuals that gave samples at all the sampling rounds, a Stata® (Stata Corp., College Station, TX) generated random subset sample, taking into account the age and sex distribution, was tested for presence of specific antibodies against cysticercosis using a commercial kit, Immunetics® (Immunetics Inc.). The assay was performed according to the manufacturer's instructions.

The stool samples, only collected during the last sampling round, were microscopically examined for *Taenia* ova using the formalin-ether concentration technique as described by Ritchie (1948) [14]. Additionally, the samples were analysed for the presence copro-antigens using a polyclonal antibody based antigen ELISA (copro-Ag-ELISA) as described by Allan *et al.* (1990) [15] with slight modifications [9].

### Prevalence and incidence calculations and statistical analyses

All collected data were entered into an excel (Microsoft Office Excel 2007®) spreadsheet and analyses were conducted in Stata 10 (Stata Corp., College Station, TX). The sampled population was distributed in 10 age categories of 10 years intervals and in function of sex.

#### Antigen and antibody seroprevalences

The antigen and antibody seroprevalences (Ag and Ab seroprevalence) were calculated for each sampling round by age category and by sex. The Two-sample test of proportion was used to compare (1) Ag/Ab seroprevalence between sex within each sampling round and (2) Ag seroprevalence with Ab seroprevalence within each sampling round. Random effect logistic regression analysis was used to compare sero-Ag and sero-Ab prevalence between rounds. Multivariate logistic regression analysis was used to study association between sero-Ag prevalence and age and sex, and this for the 3 samplings rounds. The significant level was set at 0.05. No statistical inference was done on sero-Ab prevalence and age and sex because of the low number of samples.

#### Incidence calculation

Only individuals who were sampled during all three sampling rounds were included in the incidence calculation. Individuals were defined as at risk if they were seronegative at the beginning of a period. An incident case was defined as an individual whose serology changed from negative to positive (seroconversion) between two sampling periods. Seroconversion and seroreversion was determined for both antigen and antibody results for three periods namely the first 6 months (Period 1, P1), the second 6 months (Period 2, P2) and over 12 months, that is between R1 and R3 (Period 3, P3). Seroreversion rates were calculated by dividing the number of positive tests that turned negative by the number of positive tests at the previous sampling round. Incidence rates were calculated as:

Incidence rate = number of cases in a defined time period/number of person-time units at risk during the time-period

The person-time unit represents 1 person for a defined period of time. Six monthly and yearly incidence rates were calculated first. Finally, yearly incidence rates were multiplied by 100000 to be expressed by 100000 persons-year.

The Two-sample test of proportion was used to compare (1) Ag/Ab seroconversion rates between sex within each period, and (2) Ag/Ab seroconversion rates with Ag/Ab seroreversion rates within each period. In addition, a change point analysis was used to compare Ag seroconversion rates with Ag seroreversion rates in function of age. The change point analysis classifies the population into 2 age groups at different age points (10, 20, 30, 40, 50, 60, 70, 80 years old). The Two-sample test of proportion is then used on both age groups in order to identify any change of significance when comparing Ag seroconversion and seroreversion rates. The significant level was set at 0.05.

## Results

### Sampling

A total of 3167 serum samples (from 1206 individuals from 32 villages) and 226 stool samples were examined for cysticercosis and taeniosis, respectively.

Entrees and exits of participants are explained in figure 2. A total of 1129 individuals were sampled at baseline (R1), 1069 at R2 and 969 at R3. A total of 867 (76.8%) gave samples during all sampling rounds. Reasons for lack of follow up at R2/R3 consisted of refusal to continue participating (2.7%/5.3%), away at time of sampling (3.8%/6.6%), reported sick and could not be sampled (1.2%/1.1%), died of other causes, as assessed by the RHC (0.3%/0.7%), relocated to other areas (1.2%/2.2%) and those that could not be traced (2.8%/3.1%).

From the 867 individuals sampled at R1, R2 and R3, 358 (41.3%) were men and 509 (58.7%) women; the age ranged from 2 to 87 years with a median age of 18 years. The number of people living in a HH ranged from 1 to 15 with a median of 6. From the 867 individuals that gave samples for all the sampling rounds, a random sample of 161 individuals were tested for specific antibodies against cysticercosis in each round (the same 161 participants were tested in each round).

### Household characteristics

Household characteristics (recorded from 516 HH) included; 69% of the HH kept pigs with 98% of these rearing on free-range, 46.6% of the HH did not have latrines. About 72.2% slaughter pigs in their backyards, 96.2% had at least one individual who consumed pork (boiled, fried or roasted). Only 0.6% had the meat inspected. The data obtained in the questionnaire are described in more detail in another report (Mwape *et al.*, submitted article).

### Cysticercosis and taeniosis prevalences

Table 1 shows the overall and by sex cysticercosis sero-Ag and sero-Ab prevalences per sampling.

**Table 1:** Sero-antigen and sero-antibody cysticercosis prevalences for the three sampling rounds.

Sampling round	Sex	Ag-ELISA			EITB		
		No. tested	% positive	95% CI	No. tested	% positive	95% CI
**R1**	All	1129	12.5	10.6–14.6	161	34.2	26.9–42.0
	M	464	13.6	10.6–17.0	67	32.8	22.0–45.0
	F	665	11.7	9.4–14.4	94	34.0	24.6–44.5
**R2**	All	1069	14.5	12.4–16.8	161	33.5	26.3–42.4
	M	440	16.8	13.4–20.6	67	34.3	23.2–46.9
	F	629	12.9	10.4–15.8	94	33.0	23.6–43.4
**R3**	All	969	12.2	10.2–14.4	161	38.5	31.0–46.5
	M	403	13.4	10.2–17.1	67	41.8	29.8–54.5
	F	566	11.3	8.8–14.2	94	36.2	26.5–46.7

R1, R2 and R3 stand for first, second and third round of sampling. Ag-ELISA: detection of circulating cysticercus antigen in serum; EITB: detection of specific antibodies in serum.

Sero-Ab prevalence figures (33.5–38.5%) were significantly higher than sero-Ag prevalence figures (12.2–14.5%). No significant differences were observed in sero-Ab and sero-Ag prevalences between males and females. The sero-Ab prevalence does not change between sampling rounds, the sero-Ag prevalence was significantly higher in sampling round 2 than in round 1. The probability of being sero-Ag positive increased with age for men for all three sampling rounds.

Taeniosis prevalence was determined to be 11.9% by copro-Ag-ELISA. Eleven and a half percent of the participants that tested copro-Ag positive, were also sero-Ag positive. Thirteen percent of the participants that tested copro-Ag negative, tested sero-Ag positive. *Taenia* eggs were not detected by coprological examination in any of the stool samples. Other helminth ova detected included hookworms in 20 of the samples (8.8%), *Schistosoma* spp. in 7 (3.1%) and *Trichuris trichiuria* in 2 (0.9%).

### Incidence, seroconversion and seroreversion

#### Antigen

For Period 1 (6 months), Period 2 (6 months) and Period 3 (12 months), 6.9%, 4.0% and 6.3% of the sero-Ag negative (naïve) individuals at risk of infection seroconverted, respectively; leading to a sero-Ag incidence rate of 6300 (100000 persons-year).

**Figure 3:**
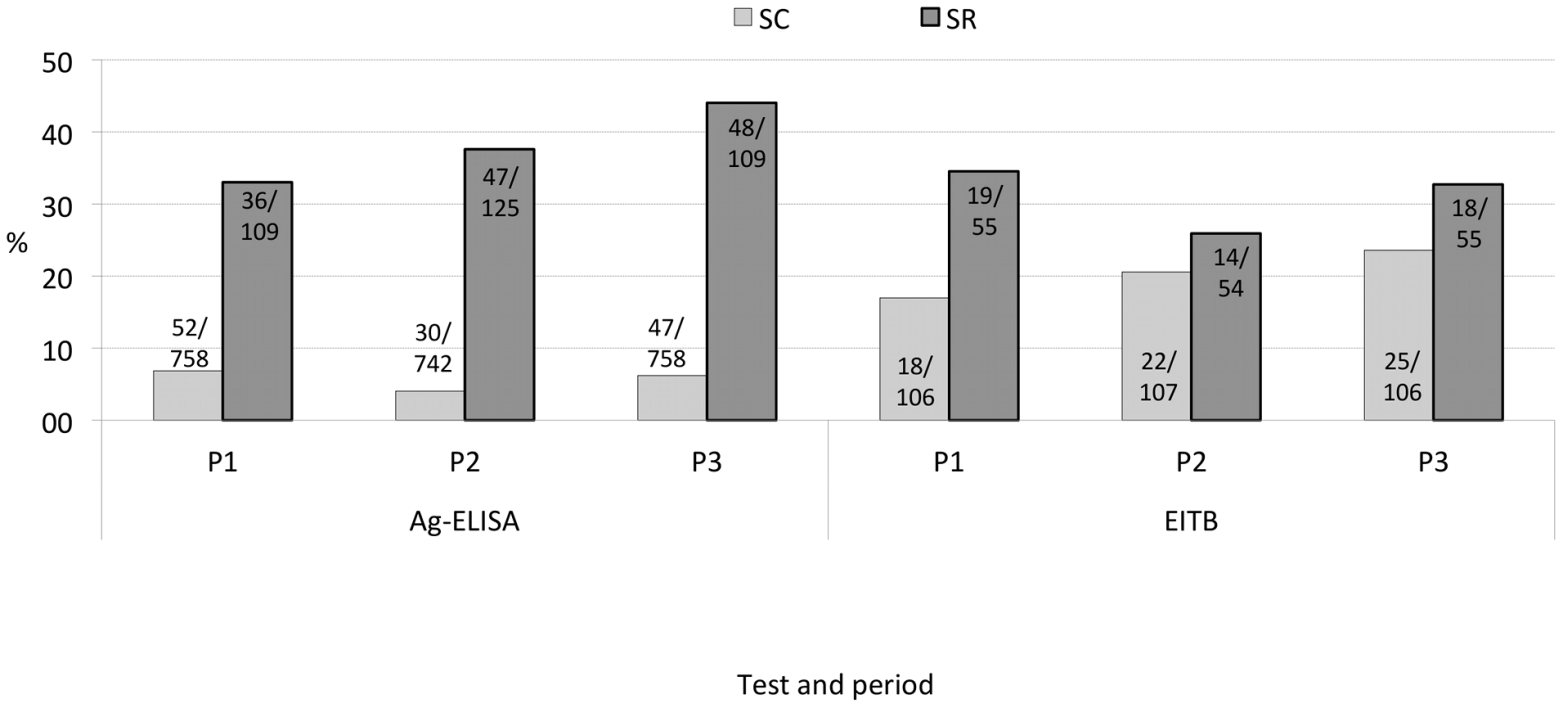
Sero- antigen and antibody conversion and -reversion for the three periods. SC and SR stand for seroconversion and seroreversion, respectively. P1 stands for period 1 (between Round 1 and Round 2, 6 months), P2 for period 2 (Round 2–3, 6 months) and P3 for period 3 (Round 1–3, 12 months). Ag-ELISA: detection of circulating cysticercus antigen in serum; EITB: detection of specific antibodies in serum.

**Figure 4:**
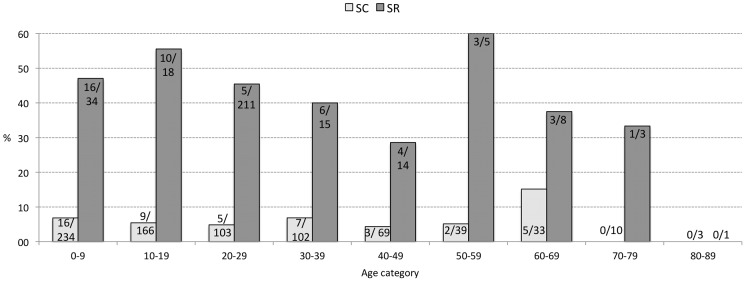
Sero-antigen conversion and -reversion in function of age categories for Period 3. SC and SR stand for seroconversion and seroreversion, respectively.

Although males recorded higher seroconversion rates than females for all periods, this was not significant (table 2). Seroreversion rates were significantly higher than seroconversion rates for each period: 36/109 (33.0%) for Period 1, 47/125 (37.6%) for Period 2 and 48/109 (44.0%) for Period 3 (Figure 3). Seroreversion rates were not significantly different between males and females. Figure 4 presents the seroconversion and -reversion rates for the different age groups for Period 3. Seroreversion was observed to be significantly higher than seroconversion in the age group up to 60 years, at which a change point was noticed. After 60 years, seroconversion rates were not significantly different from seroreversion rates.

**Table 2:** Seroconversion rates of human cysticercosis in function of sex for both circulating antigen and specific antibody analyses.

Period	Sex	New cases	Individuals at risk	Seroconversion (%)	95% CI
**Ag-ELISA**					
P1	All	52	758	6.9	5.2–8.9
	M	26	331	7.9	5.2–11.3
	F	26	447	5.8	3.8–8.4
P2	All	30	742	4.0	2.7–5.7
	M	14	297	4.7	2.6–7.8
	F	16	445	3.6	2.1–5.8
P3	All	47	758	6.3	4.6–8.2
	M	24	311	7.7	5.0–11.3
	F	23	443	5.2	3.3–7.7
**EITB**					
P1	All	18	106	17.0	10.4–25.5
	M	11	51	21.6	11.3–35.3
	F	7	55	12.7	5.3–24.5
P2	All	22	107	20.6	13.4–29.4
	M	11	42	26.2	13.9–43.8
	F	11	65	16.9	8.8–28.3
P3	All	25	106	23.6	15.9–32.8
	M	15	51	29.4	17.5–43.8
	F	10	55	18.2	9.1–30.9

P1 stands for period 1 (between Round 1 and Round 2, 6 months), P2 for period 2 (between Round 2 and 3, 6 months) and P3 for period 3 (between Round 1 and 3, 12 months). Ag-ELISA: detection of circulating cysticercus antigen in serum; EITB: detection of specific antibodies in serum.

#### Antibody

For Period 1, 2 and 3, 17%, 20.6% and 23.6% of the sero-Ab negative (naïve) individuals at risk of infection seroconverted (Table 2), leading to a sero-Ab incidence rate of 23600 (100000 persons-year). As for sero-Ag, the differences in seroconversion between males and females were not significant. Antibody seroreversion (34.5% for P1, 25.9% for P2 and 32.7% for P3) was higher than seroconversion, although only significant in P1 (Figure 3). Seroreversion rates were not different between males and females for P1 and P2.

Table 3 shows the infection dynamics, as measured by antibody and antigen presence, throughout the three sampling rounds. A high percentage (78.4%) of individuals remained sero-Ag negative throughout the study, compared to 44.7% for sero-Ab. Almost 7% (6.6%) and about 19% of the individuals remained sero-Ag and sero-Ab positive throughout the study, respectively. Thirty one of 867 (3.6%) (sero-Ag) and 9 of 161 (5.6%) (sero-Ab) of the participants were seronegative at the start of the survey, became seropositive at R2 and turned negative again by R3. Vice versa, 0.5% (sero-Ag) and 3.7% (sero-Ab) of the participants tested were seropositive at R1, became seronegative at R2 and again seropositve at R3.

**Table 3:** Infection/exposure status changes based on sero-antigen and sero-antibody analysis throughout the study period.

Status per sampling round			Test			
			Ag-ELISA		EITB	
R1	R2	R3	No.	%	No.	%
**N**	**N**	**N**	680	78.4	72	44.7
**N**	**N**	**P**	26	3.0	16	9.9
**N**	**P**	**P**	21	2.4	9	5.6
**N**	**P**	**N**	31	3.6	9	5.6
**P**	**N**	**N**	32	3.7	13	8.1
**P**	**N**	**P**	4	0.5	6	3.7
**P**	**P**	**N**	16	1.8	5	3.1
**P**	**P**	**P**	57	6.6	31	19.3

R1, R2 and R3 stand for first, second and third rounds of sampling. N and P stand for negative and positive test result, respectively. Ag-ELISA: detection of circulating cysticercus antigen in serum. “No.” expresses the number of participants that presented the given statuses (e.g. N N N) during the follow up (e.g. 680 participants tested negative at all three sampling rounds, N N N); EITB: detection of specific antibodies in serum “No.” expresses the number of participants that presented the given statuses during the follow up (e.g. 72 participants of the 161 tested negative at all three sampling rounds, N N N. This number is independent of the Ag-ELISA result).

## Discussion

The present study is the first to estimate the incidence of human cysticercosis based on specific antibody as well as antigen detections; adding to the very short list of publications reporting the incidence of human cysticercosis [16]. The high taeniosis prevalence (11.9%) in this study is strongly indicative for a high environmental contamination with *T. solium* eggs, and subsequent high exposure risk. The high sero-antibody results (33.8–38.5% sero-Ab prevalence) as well as the fact that less than half of the sampled population (44.7%) remained negative (sero-Ab) throughout the study period corroborate this finding, as presence of specific antibodies is indicative for exposure to infection [17]. About 32% (34/106) of the participants negative at the start of the study turned Ab positive at one point; an additional 6 tested participants positive at R1, but negative at R2, turned positive again at R3 (table 3), indicating that more than one on three people have been (re) exposed and reacted to infection during the study period.

The sero-Ag results present a different picture. A much higher percentage (78%) of people remained negative throughout the study; and only 11.5% of the participants negative at the start of the study turned positive at one point (table 3). As presence of antigen indicates establishment of infection rather than exposure, these results strongly indicate that about one on three people are exposed to infection, whereas the infection only establishes in about one on ten people. Findings from studies in Peru in pigs and human and in Ecuador in human [11] also suggest exposure without infection or mild infections that are aborted by the natural immunity of the individual, expressed by the presence of transient antibodies [18]. The higher levels of sero-Ab prevalence and seroconversion in comparison with sero-Ag prevalence/conversion, as well as the high seroreversion levels, identified in this study, support this finding.

Another interesting outcome from this study is the rather short-term presence of antigen in 31 participants (negative at R1, positive at R2, and again negative at R3, table 3). Whether this is due to an only partial establishment of infection (immature cysticerci), or establishment and quick degeneration (self cure?) of cysticerci is not clear. It was noted that individuals who became seronegative were those with samples that had low antigen titers (Data not shown). In humans, it is described that cysticerci in the brain usually stay viable during years, while probably cysticerci in the muscles tend to degenerate more quickly [19]. However later, Garcia *et al.* (2010) [20] challenged this theory in the case of single cysticercal granuloma's, for which they hypothesize that instead of being caused by a late degenerative process, the granulomas are rather due to an early parasite death. In experimental infections in pigs often infections do not establish, or (partially) establish (with the corresponding increase in antigen levels) and abort shortly afterwards. Deckers *et al.*, (2008) [21] indeed demonstrated circulating cysticercus antigens as early as three weeks after experimental infection, which is before full maturation of the cysticerci. Many factors, among which the size of the (re) infection, the immune status of the host, age and sex play a determining role in the (non) establishment of infection [22]. Results from this study suggest that presence of antigen doesn't necessarily always signify presence of a viable, well established infection, however could be indicative for short term partial establishment, and perhaps a ‘transient’ antigen presence should be considered. As such, serological results from field studies, should be looked at critically. Individuals with positive test results shouldn't be automatically considered as ‘infected with *T. solium*’, as is often done in reports from field studies.

Significantly higher sero-Ag reversion than seroconversion was determined up to the age of 60 years. Previous studies have indicated higher levels of active infection in elderly people, which was suggested to be due to a lowered host immune response [11]. The higher seroreversion rates than seroconversion rates observed in younger people, but not in older people in this study, could indeed be indicative of an improved clearing of the infection in younger people. The simulation models described in Praet *et al.* (2010) [11] suggest a continuous exposure of the population with seroreversion (antibody) rates depending on the number of exposures, which relates to age as well as the immunological status of the individual. Antibody seroreversion rates of 60% after first exposure and 20% after second and subsequent exposures were obtained.

This is the first study to report cysticercosis incidence based on sero-Ag analysis (6300 per 100000 persons-year). The sero-Ab incidence rate (23600 per 100000 persons-year) is comparable to that reported in Peru by Garcia *et al*. (2001) [10] and in Ecuador [11]. A higher average porcine cysticercosis sero-Ab incidence rate of 53% has been reported in Peru [18]. Since pigs are highly coprophagic, it is expected that they would be exposed more frequently and to higher levels of infection as compared to humans and hence record a higher incidence rate especially for sero-Abs.

In conclusion, this study has shown the dynamic nature of *T. solium* infections, many of the people at risk become (re)infected due to the high environmental contamination, with a high number turning seronegative within a year after infection. An important number of infections probably never fully establish, leading to transient antibody responses and possibly even ‘transient’ antigen presence.

## Supporting Information

Checklist S1 **Strobe checklist.**
(PDF)

## References

[1] Del Brutto OH, Rajshekhar V, White AC Jr, Tsang VC, Nash TE, et al (2001) Proposed diagnostic criteria for neurocysticercosis. Neurology 57: 177–183. 11480424 10.1212/wnl.57.2.177 PMC2912527

[2] Parija SC, Sahu PS, Dhanya H (2007) Detection of Cysticercus antigens and antibodies in cerebrospinal fluid of patients with chronic meningitis. Revista do Instituto de Medicina Tropical de Sao Paulo 49: 331–334. 18026642 10.1590/s0036-46652007000500011

[3] Murrell KD, editor (2005) WHO/FAO/OIE Guidelines for the Surveillance, Prevention and Control of Taeniosis/Cysticercosis. Paris (France): OIE.

[4] Ndimubanzi PC, Carabin H, Budke CM, Nguyen H, Qian YJ, et al (2010) A systematic review of the frequency of neurocyticercosis with a focus on people with epilepsy. PLoS Negl Trop Dis 4: e870. 21072231 10.1371/journal.pntd.0000870 PMC2970544

[5] Torgerson PR, Macpherson CN (2011) The socioeconomic burden of parasitic zoonoses: global trends. Veterinary parasitology 182: 79–95. 21862222 10.1016/j.vetpar.2011.07.017

[6] Kanobana K, Praet N, Kabwe C, Dorny P, Lukanu P, et al (2011) High prevalence of *Taenia solium* cysticerosis in a village community of Bas-Congo, Democratic Republic of Congo. International journal for parasitology 41: 1015–1018. 21763695 10.1016/j.ijpara.2011.06.004

[7] Sacks LV, Berkowitz I (1990) Cysticercosis in an urban black South African community: prevalence and risk factors. Tropical gastroenterology : official journal of the Digestive Diseases Foundation 11: 30–33. 2356575

[8] Vilhena M, Santos M, Torgal J (1999) Seroprevalence of human cysticercosis in Maputo, Mozambique. The American journal of tropical medicine and hygiene 61: 59–62. 10432057 10.4269/ajtmh.1999.61.59

[9] Mwape KE, Phiri IK, Praet N, Muma JB, Zulu G, et al (2012) *Taenia solium* Infections in a rural area of Eastern Zambia-a community based study. PLoS neglected tropical diseases 6: e1594. 22479664 10.1371/journal.pntd.0001594 PMC3313923

[10] Garcia HH, Gonzalez AE, Gilman RH, Palacios LG, Jimenez I, et al (2001) Short report: transient antibody response in *Taenia solium* infection in field conditions-a major contributor to high seroprevalence. Am J Trop Med Hyg 65: 31–32. 11504404 10.4269/ajtmh.2001.65.31

[11] Praet N, Speybroeck N, Rodriguez-Hidalgo R, Benitez-Ortiz W, Berkvens D, et al (2010) Age-related infection and transmission patterns of human cysticercosis. International journal for parasitology 40: 85–90. 19683531 10.1016/j.ijpara.2009.07.007

[12] Sikasunge CS, Phiri IK, Phiri AM, Siziya S, Dorny P, et al (2008) Prevalence of *Taenia solium* porcine cysticercosis in the Eastern, Southern and Western provinces of Zambia. Vet J 176: 240–244. 17468023 10.1016/j.tvjl.2007.02.030

[13] Dorny P, Phiri IK, Vercruysse J, Gabriel S, Willingham AL 3rd, et al (2004) A Bayesian approach for estimating values for prevalence and diagnostic test characteristics of porcine cysticercosis. Int J Parasitol 34: 569–576. 15064121 10.1016/j.ijpara.2003.11.014

[14] Ritchie LS (1948) An ether sedimentation technique for routine stool examinations. Bulletin of the US Army Medical Department United States Army Medical Dept 8: 326. 18911509

[15] Allan JC, Avila G, Garcia NJ, Flisser A, Craig PS (1990) Immunodiagnosis of taeniasis by coproantigen detection. Parasitology (3): 473–477. 10.1017/s0031182000060686 2092303

[16] Villaran MV, Montano SM, Gonzalvez G, Moyano LM, Chero JC, et al (2009) Epilepsy and neurocysticercosis: an incidence study in a Peruvian rural population. Neuroepidemiology 33: 25–31. 19325247 10.1159/000210019 PMC2826439

[17] Praet N, Rodriguez-Hidalgo R, Speybroeck N, Ahounou S, Benitez-Ortiz W, et al (2010) Infection with versus exposure to *Taenia solium*: what do serological test results tell us? Am J Trop Med Hyg 83: 413–415. 20682891 10.4269/ajtmh.2010.10-0121 PMC2911194

[18] Garcia HH, Gonzalez AE, Gavidia C, Falcon N, Bernal T, et al (2003) Seroincidence of porcine *T. solium* infection in the Peruvian highlands. Preventive veterinary medicine 57: 227–236. 12609467 10.1016/s0167-5877(02)00234-9

[19] Garcia HH, Del Brutto OH (2005) Neurocysticercosis: updated concepts about an old disease. Lancet Neurol 4: 653–661. 16168934 10.1016/S1474-4422(05)70194-0

[20] Garcia HH, Gonzalez AE, Rodriguez S, Tsang VC, Pretell EJ, et al (2010) Neurocysticercosis: unraveling the nature of the single cysticercal granuloma. Neurology 75: 654–658. 20713953 10.1212/WNL.0b013e3181ed9eae PMC2931772

[21] Deckers N, Kanobana K, Silva M, Gonzalez AE, Garcia HH, et al (2008) Serological responses in porcine cysticercosis: A link with the parasitological outcome of infection. Int J Parasitol (10): 1191–1198. 10.1016/j.ijpara.2008.01.005 18328486

[22] Fleury A, Dessein A, Preux PM, Dumas M, Tapia G, et al (2004) Symptomatic human neurocysticercosis–age, sex and exposure factors relating with disease heterogeneity. J Neurol 251: 830–837. 15258785 10.1007/s00415-004-0437-9

